# Intraocular Pressure Measurements in Standing, Sitting, Prone, and Supine Positions

**DOI:** 10.3390/jpm14080826

**Published:** 2024-08-04

**Authors:** Maddalena De Bernardo, Ferdinando Cione, Ilaria De Pascale, Sergio Pagliarulo, Nicola Rosa

**Affiliations:** Eye Unit, Department of Medicine Surgery and Dentistry, “Scuola Medica Salernitana”, University of Salerno, 84081 Salerno, Italy; mdebernardo@unisa.it (M.D.B.);

**Keywords:** intraocular pressure, hand-held tonometer, standing position, supine position, sitting position, normal-tension glaucoma

## Abstract

In this study, intraocular pressure (IOP) was measured in sitting, supine, prone, and standing (ST) positions and again five minutes after standing (ST-5) utilizing a Tono-Pen AVIA in 124 eyes of 62 healthy subjects with ages ranging from 21 to 59 years (mean 30 ± 10 years). In each subject, the average IOP of both eyes was used for the statistical evaluation. The mean IOP difference between the ST and sitting positions was −0.13 ± 1.63 mmHg (*p* = 0.548); between ST-5 and sitting, it was 0.53 ± 1.24 mmHg (*p* = 0.001); between supine and sitting, it was 1.30 ± 1.48 mmHg (*p* < 0.001); between ST and supine, it was −1.43 ± 1.74 mmHg (*p* < 0.001); between ST-5 and supine, it was −0.77 ± 1.59 mmHg (*p* < 0.001); between prone and supine, it was 2.24 ± 1.92 mmHg (*p* < 0.001); between ST and ST-5, it was −0.67 ± 1.84 mmHg (range: −7.5 to 5 mmHg) (*p* = 0.007); between prone and ST, it was 3.46 ± 2.01 mmHg (*p* < 0.001); between ST-5 and prone, it was −2.46 ± 1.67 mmHg (*p* < 0.001); and between sitting and prone, it was −3.22 ± 1.56 mmHg (*p* < 0.001). The results show a significant IOP increase in the ST-5 position, suggesting that such measurements need to be performed in an attempt to explain the progression of glaucoma in apparently normal-tension patients.

## 1. Introduction

The increase in intraocular pressure (IOP) is a well-established risk factor for glaucoma development and progression [1,2,3]. Given improvements in understanding glaucoma pathophysiology, IOP remains the only variable that therapy can improve [4]. For this reason, IOP measurement is part of routine ophthalmic examinations. Unfortunately, these examinations represent only a snapshot of the real IOP due to its circadian fluctuations that may influence glaucoma progression [5,6,7].

Among the variables that can influence this fluctuation, the position of the body can play a major role in nocturnal IOP elevation; in fact, several studies have demonstrated that the IOP may rise significantly when the patient assumes a supine position compared to a sitting position [8,9,10]. In some cases, this could explain why glaucomatous damage continues to progress despite the detection of a low IOP during routine ophthalmic examinations, suggesting that some high-risk patients should sleep with their head elevated [11]. It should be taken in account that many people can spend hours in a standing position during the day, and for this reason, it could be helpful to know if pressure changes occur in this situation. In a PubMed literature search, it was possible to find just four papers that reported some information about this topic [12,13,14,15]. Among these, in one paper, IOP measurements taken in a standing position were compared with those taken an unusual position, suspending the patients by the ankles from a gravity inversion device [12], while another study was performed on a very limited number of patients [13], and two others used rebound tonometers (Icare Pro–ICP) [14,15]. 

The rebound principle upon which Icare is based is different from the applanation principle of Goldmann applanation tonometry (GAT), which is still considered the gold standard in IOP measurement. Also, Tono-Pen AVIA (TPA) uses the same physical principle to measure the IOP, but it has two advantages:

(1) The applanated area is much smaller, because the transducer tip has a diameter of 1.0 mm.
(2) TPA is portable and it does not need to be installed on a slit-lamp, and therefore it can be used to measure IOP in different IOP positions [16].

A previous paper published by De Bernardo et al. evaluated TPA and rebound tonometers in different body positions, but they carried out their study on a small number patients and not all body positions were analyzed [16]. On these bases, in continuity with previously published evidence, the aim of this study was to evaluate IOP measurements performed with an applanation-based tonometer by checking if similar changes were observed in a larger population after 5 min or more in a standing position and in a prone position.

## 2. Materials and Methods

One hundred and twenty-four eyes of 62 healthy subjects (16 males) with an age ranging from 21 to 59 years old (mean 30 ± 10 years) and a refractive error ranging from −6.50 to +1.25 D (mean −2.07 ± 2.13 D) were included in this prospective observational study. 

The study was conducted in adherence to the tenets of the World Medical Association’s Declaration of Helsinki. The purpose of the study was explained to all participants, who provided written informed consent. The study was approved by the Institutional Review Board of the University of Salerno (Cometico Campania Sud, Naples., Italy, Protocol No. 16544). Each subject underwent a general physical checkup to rule out any systemic disease. 

The participants underwent a comprehensive ophthalmic examination that included the following:

- Best corrected visual acuity;
- Refractive error, performed with an objective method and perfected with a subjective method;
- Fundus examination;
- Axial eye length (AL), evaluated with an IOLMaster (Zeiss, Jena, Germany, version 5.4.4.00006);
- Central corneal thickness (CCT) measured with a Pentacam HR (Oculus, Wetzlar, Germany, version 1.19r11). IOP measurements were taken with TPA (Reichert Inc., Depew, NY, USA).

IOP was measured between ten and twelve a.m. by a single observer. TPA used the same physical principle as GAT to measure IOP, but the applanated area is much smaller. Before each measurement, the eyes were anesthetized with oxibuprocaine eye drops and the TPA probe tip was covered with a new latex tip cover. When ten valid readings were obtained by slightly touching the central cornea, the mean IOP readings were automatically averaged by the instrument. The measurement was shown on the liquid crystal display, situated on the device side, together with the “statistical confidence indicator”, indicating that the standard deviation of the valid measurements was 5% or less of the number shown: the higher the value, the more reliable the measurements [16]. Only values higher than 90 were accepted.

The measurements were obtained in a sitting position, a supine position after five minutes of lying down, a prone position, a standing (ST) position, and after five minutes in a standing position (ST-5) in a random sequence [14]. The measurements in a prone position were taken with the patient’s head protruding beyond the bed, with the chin resting on the bed and the eyes looking downwards. Unfortunately, not all patients were able to position their body to perform correct IOP measurements in a prone position: for this reason, prone measurements were taken in 23 patients. In addition, in 4 patients, the IOP measurements in an ST position were not performed. In each patient, the average of both eyes’ IOP was analyzed using the paired Student’s *t*-test. Single pairwise comparisons through T-tests were preferred to repeated-measures ANOVA tests because of the different sample sizes available. Correlations analyses were performed with Pearson’s test (r: correlation coefficient according to Pearson). All statistical analyses were performed with Microsoft Excel software, and they were double-checked with SPSS software (Version 26.0 IBM Inc., Chicago, IL, USA). Data were expressed as means ± standard deviations and *p* values < 0.050 was considered significant. The preliminary required sample size was computed with G*Power software (Version 3.1.9.7) by using the T test family option, two-tailed. Given an effect size dz of 0.786, determined with G*Power software, it was estimated that a sample size of 20 subjects would be necessary, with a significance level of 0.05 and a test power of 0.90.

## 3. Results

**Figure 1:**
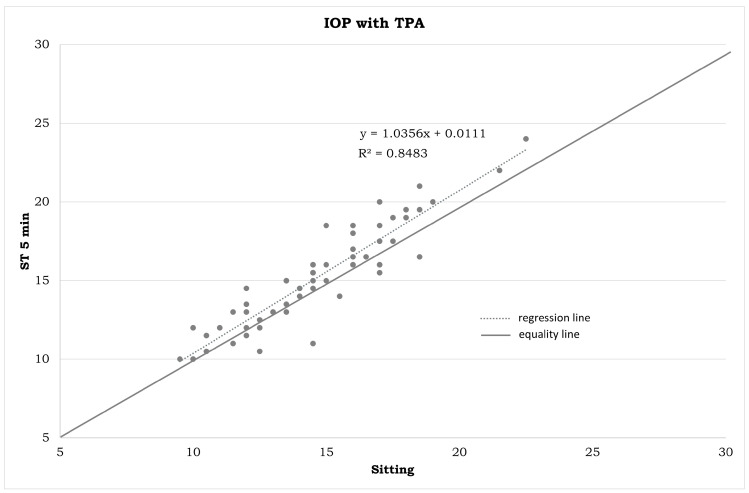
Plot between the IOP measurements in a sitting position on the horizontal axis and IOP measurements in the ST-5 min position on the vertical axis (in mmHg). IOP: intraocular pressure. TPA: Tono-Pen AVIA. ST-5 min: measurements obtained after five minutes in a standing position.

**Figure 2:**
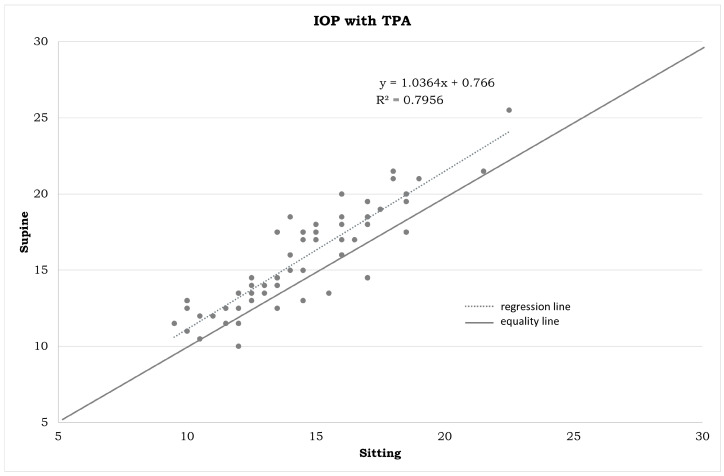
Plot between the IOP measurements in a sitting position on the horizontal axis and IOP measurements in a supine position on the vertical axis (in mmHg). IOP: intraocular pressure. TPA: Tono-Pen AVIA.

**Figure 3:**
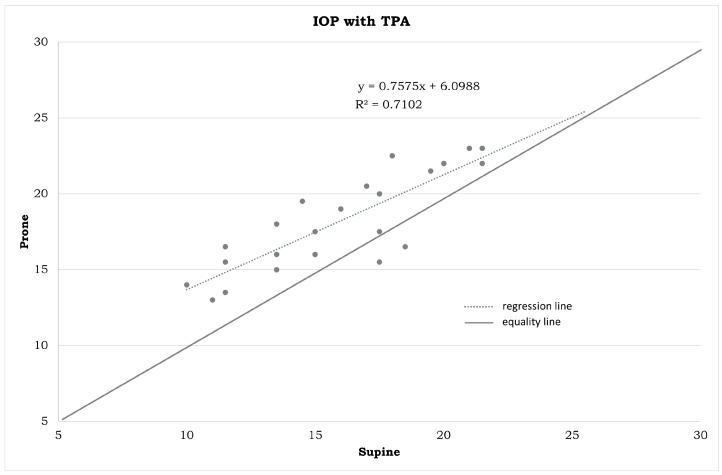
Plot between the IOP measurements in a supine position on the horizontal axis and IOP measurements in a prone position on the vertical axis (in mmHg). IOP: intraocular pressure. TPA: Tono-Pen AVIA.

**Figure 4:**
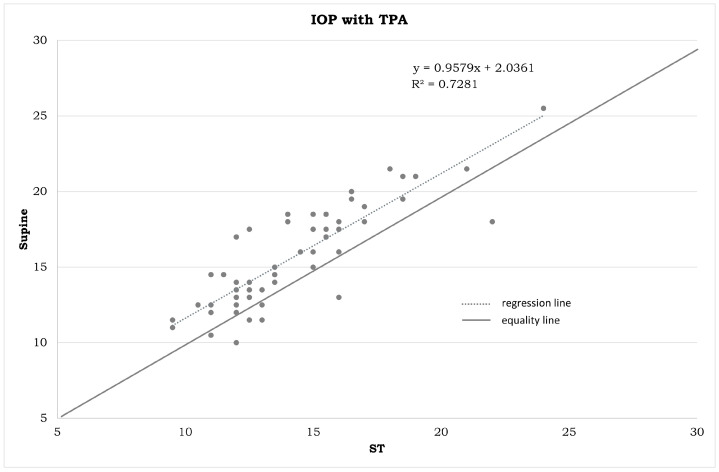
Plot between the IOP measurements in an ST position on the horizontal axis and IOP measurements in a supine position on the vertical axis (in mmHg). IOP: intraocular pressure. TPA: Tono-Pen AVIA. ST: measurements obtained immediately after assuming a standing position.

**Figure 5:**
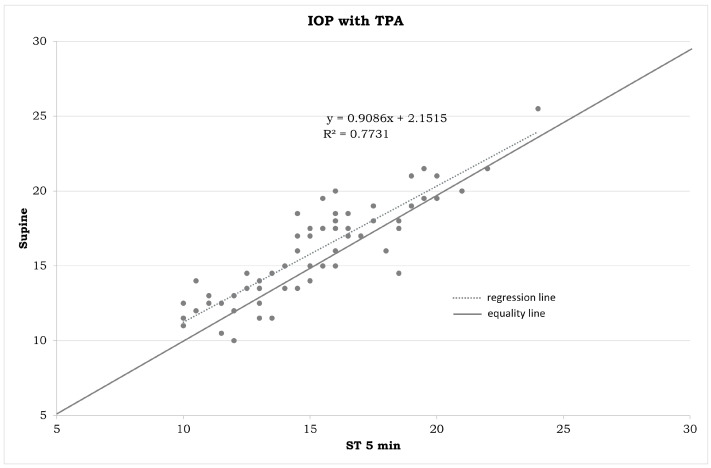
Plot between the IOP measurements in the ST-5 min position on the horizontal axis and IOP measurements in a supine position on the vertical axis (in mmHg). IOP: intraocular pressure. TPA: Tono-Pen AVIA. ST-5 min: measurements obtained after five minutes in a standing position.

**Figure 6:**
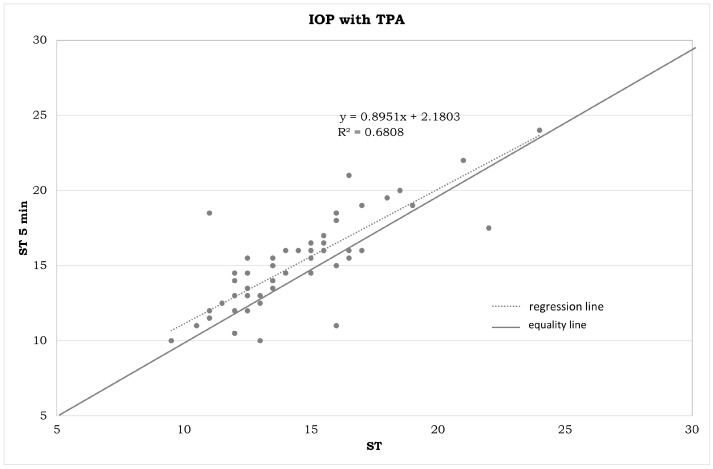
Plot between the IOP measurements in a ST position on the horizontal axis and IOP measurements in the ST-5 min position on the vertical axis (in mmHg). IOP: intraocular pressure. TPA: Tono-Pen AVIA. ST-5 min: measurements obtained after five minutes in a standing position. ST: measurements obtained immediately after assuming a standing position.

IOP measurements in the different positions (Table 1) and the statistical differences summarized in Figure 1, Figure 2, Figure 3, Figure 4, Figure 5, Figure 6, Figure 7, Figure 8 and Figure 9 and Table 2 clearly show that:

- In the ST-5 and supine positions, the IOP is higher than in the sitting position (*p* < 0.001) in both cases (Figure 1 and Figure 2, correspondingly);
- In the prone position, the IOP is higher than in the supine position (*p* < 0.001, Figure 3);
- In the supine position, the IOP is higher than in the ST position (*p* < 0.001, Figure 4), and this difference decreases with increasing time (ST-5 min, Figure 5);
- In the ST-5 min position, the IOP is higher than in the ST position (*p* = 0.007, Figure 6).

The CCT measured with the Pentacam HR was 535 ± 32 µm (range: 452 to 598 µm).

The AL obtained from the IOLMaster was 24.47 ± 1.28 mm (range: 21.70 to 27.16 mm).

**Figure 7:**
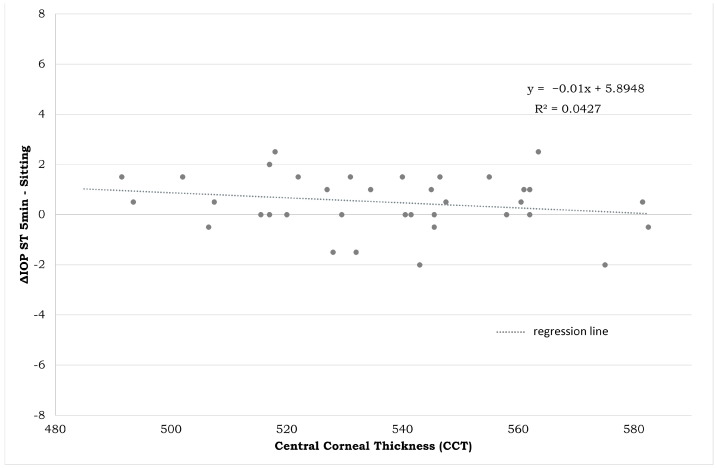
Plot between the CCT mean of both eyes of each subject on the horizontal axis (in micron) and difference in IOP measurements between the ST-5 and sitting positions on the vertical axis (in mmHg). CCT: central corneal thickness. IOP: intraocular pressure. ST-5 min: measurements obtained after five minutes in a standing position.

**Figure 8:**
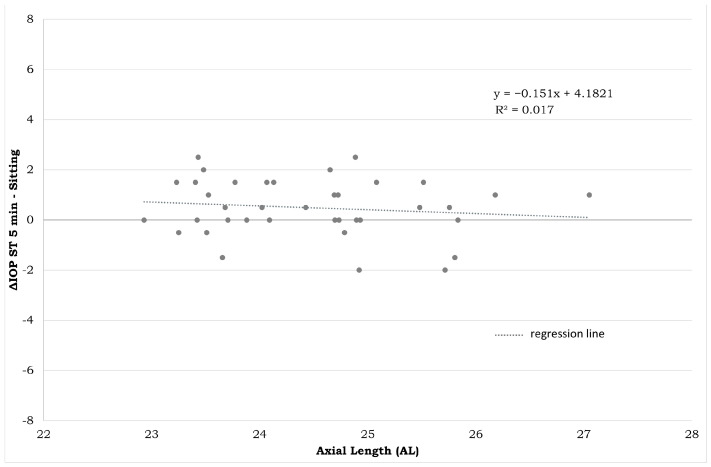
Plot between the AL mean of both eyes of each subject on the horizontal axis (in mm) and difference in IOP measurements between the ST-5 and sitting positions on the vertical axis (in mmHg). IOP: intraocular pressure. ST-5 min: measurements obtained after five minutes in a standing position.

IOP differences in the sitting and ST-5 min positions presented no correlation with CCT (Figure 7) and AL (Figure 8).

**Figure 9:**
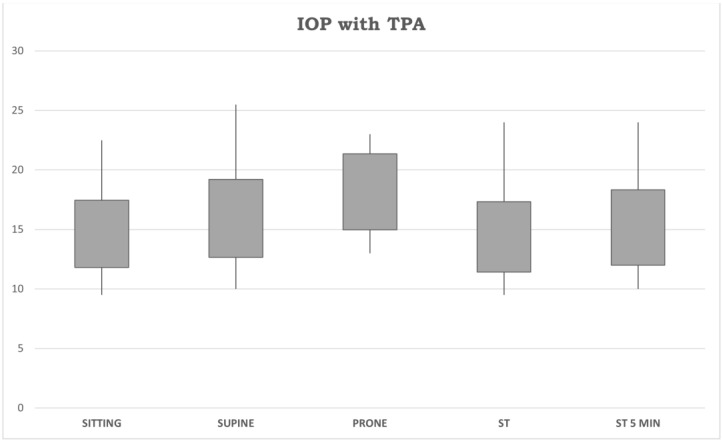
Standard deviations and minimum/maximum values for IOP measurements in the sitting, supine, prone, ST and ST-5 min positions (in mmHg). IOP: intraocular pressure. TPA: Tono-Pen AVIA. ST 5 MIN: measurements obtained after five minutes in a standing position. ST: measurements obtained immediately after assuming a standing position.

Means and standard deviations for IOP measurements in the sitting, supine, prone, ST, and ST-5 min positions (in mmHg) are shown in Figure 9.

### Figures and Tables 

Table 1 synthesizes the IOP values obtained with the TPA in all positions, while Table 2 shows the mean differences between IOPs measured in all positions, with the level of significance. We should reiterate that comparisons with ST data were performed based on 58 patients; meanwhile, analysis with prone position data were carried out on 23 patients.

Figure 1, Figure 2, Figure 3, Figure 4, Figure 5 and Figure 6 represent the correlation analysis between IOP values in different body positions. A strong positive correlation was found

- between sitting and ST-5 IOP (r = 0.921);
- between sitting and supine IOP (r = 0.891);
- between supine and prone IOP (r = 0.843);
- between ST and supine IOP (r = 0.853);
- between ST-5 and supine IOP (r = 0.879);
- between ST and ST-5 IOP (r = 0.825).

For brevity, only the most clinically relevant correlations were reported.

The correlations between biometric parameters and IOP changes are shown in Figure 7 and Figure 8. A low correlation rate was found between both AL and CCT and ∆IOP ST-5/sitting (r: −0.330 and r: −0.439, respectively), meaning that biometric parameters are less involved in IOP changes across different body positions. For brevity, only the most clinically relevant correlations were reported. Figure 9 reports graphically descriptive statistic data of IOP values in different body positions.

A cluster analysis based on patients’ age was carried out in order to verify personalized responses. Due to the low number of prone measurements, these data were not considered in cluster analysis to avoid a too low sample size. The median age of subjects (25 years) was considered as the cut-off value for age range analysis. Overall, 35 subjects (31 in standing position) were evaluated in subgroup A (patients’ age ≤ 25 years), while 27 subjects were evaluated in subgroup B (patients’ age > 25 year). The results are reported in Table 3. 

The same trend was noted both in the general population and in the age-divided subgroups for supine/sitting and ST/supine. The difference in ST/sitting was not statistically significant in all groups, and instead a lower difference for ST-5/sitting was found in younger patients and a lower difference for ST-5/supine and ST/ST-5 was found in older patients.

## 4. Discussion

Goldmann applanation tonometry (GAT) is considered to be the gold standard in IOP measurements, but unfortunately it has several limitations, including that it needs to be mounted on a slit lamp, and for this reason, it is almost impossible to use in patients in a supine or a standing position; for this reason, this study was conducted with TPA, which has been reported to give reliable measurements compared to GAT in a sitting position [17]. 

In a PubMed literature search, it was possible to find several papers where the IOP was measured in sitting and supine position with different devices, some with the Tonopen [18,19,20,21,22], some others with the Icare [23], others with both tonometers [24,25,26], and only one with TPA [24]. However, just four papers investigated the IOP in a standing position, and they made different comparisons [12,13], or the authors used an ICP [14,15].

LeMarr et al. [12], in a group of 26 young subjects, evaluated the IOP changes between a standing and a head-down position. They found an increase in IOP in the head-down position, which a very unusual position; moreover, no comparison was made with a sitting or supine position. 

Singleton et al. [13] evaluated the changes in IOP with postural changes (supine, sitting, and standing) in just 11 normal subjects, comparing them to the changes that occur in several groups of patients with different kinds of autonomic dysfunction. In this very small group of normal subjects, they found that the changes in IOP with postural changes were not statistically significant.

Meanwhile, some studies have measured the IOP disparity between sitting and supine postures. Lee et al. [17] examined 19 healthy young Korean participants, comparing the IOP in a sitting and supine position with a Tonopen XL: they observed an increase of approximately 3 mmHg when moving from a sitting to a supine position.

In another study, Lee et al. [23] measured the IOP in 40 eyes of 20 healthy Korean subjects using an Icare tonometer and found a statistically significant mean increase in IOP (*p* < 0.001) of about 2 mmHg when shifting from a sitting to a supine position. 

Moster et al. [20] measured the IOP in both a sitting and a supine position in 45 glaucoma patients and 46 healthy subjects with a Tonopen. In each group, the authors found a statistically significant mean IOP increase from a sitting to a supine position, being higher in glaucomatous patients (2.3 mmHg) than in healthy subjects (1.2 mmHg).

Barkana et al. tested the IOP with a Tonopen XL in 19 healthy subjects [21], first sitting then lying for 15 ± 5 min and 45 ± 5 min in a supine position; the same authors in another study measured the IOP with a Tonopen XL in twenty-one eyes of 21 healthy subjects [24], first in a sitting position and then after 10 min in a supine position. In both studies, the authors found an increase in IOP from a sitting to a supine position; furthermore, in the first study, the IOP after 45 min in a supine position was less than that measured after 15 min, without statistically significant differences.

Schweier et al. [25], in 36 eyes of 36 healthy individuals, measured IOP with ICP, TPA, and GAT in a sitting position and 10 min after reclining. They found the IOP to be lower in the sitting position compared to the reclining position with both hand-held tonometers, and the mean difference was greater with TPA (1.8 mmHg) than with ICP (0.8 mmHg) [25]. 

Sobczak et al. [26], in 71 healthy subjects, found no statistically significant differences between IOP measurements in sitting and supine positions using an ICP tonometer. 

Our results regarding the IOP changes in a standing position cannot be compared with the previously published papers by LeMarr [12] and Singleton [13], due to the lack of measurements in the sitting position in the first case and the limited number of patients in the second case.

The results of the present study agree with those published by De Bernardo et al. [14], indicating that utilizing the ICP revealed a non-significant increase in the ST position compared to the sitting position, followed by a statistically significant increase in the ST-5 min position compared to the ST position. Mayali et al. [15], on the other hand, using rebound tonometry (ICP), found no IOP differences in the standing position versus other body positions.

Compared with other papers that considered only measurements in sitting and supine positions, the present study, performed with TPA, confirms these findings. However, our finding of a significant increase in the IOP in a prolonged standing position could be an interesting new finding, even if it is less than the increase in a supine position. 

The IOP increase in the supine position may have different explanations; in particular, some researchers have indicated that this is due to an increase, in the supine position, in the episcleral venous pressure [27]. Other authors suggest that this could be due to choroidal vascular engorgement caused by the redistribution of bodily fluids in the supine position [28]. 

The results displayed in Figure 7 and Figure 8 clearly show that the IOP measured in the different postures is not related to the CCT or to the AL. The reason for the increase in prolonged standing position is not as obvious. One hypothesis could be that, even if the exact mechanism of the IOP regulation is unknown, it may be under systemic vascular control, through the autonomous nervous system, which contributes to maintaining relatively constant blood flow to the tissue despite perfusion pressure fluctuations. Local myogenic, metabolic, and circulating humoral factors are involved in the autoregulation process [13,29]. However, future studies correlating the postural changes in IOP with eventual changes in blood pressure, heart rate, ocular perfusion pressure, and peripheral vasoconstriction could be useful to better understand the reason for these IOP modifications. 

Although the sample in our study is not representative for all age groups, previous studies suggest that the IOP change associated with different body positions is not age-related [15]; a cluster analysis based on patients’ age was carried out in order to verify if the same trend in IOP changes persists in different age ranges. As reported in Table 3, younger subjects showed a lower difference between the ST-5 and sitting positions than the general population; on the other hand, differences between ST-5 and both the supine and ST positions were lower in older patients. A more valid vascular flow redistribution could explain the lower difference between the first (sitting position) and the last (ST-5 position) IOP measurements in younger patients. On the other hand, a more stable choroidal vascular engorgement could explain the lower differences between ST-5 and both the ST and supine positions in older subjects. These findings could help in a personalized analysis of IOP changes in normal subjects and glaucomatous patients, according to their age range. Unfortunately, due to only male subjects being examined in this study, a gender-based analysis of IOP changes in different positions was inexecutable.

One criticism that could be applied to this study is that the TPA was used instead of the GAT, which, introduced in 1957 by Hans Goldmann, represents the gold standard in measuring IOP [30]. Unfortunately, it is well known that the measured IOP value is influenced by several corneal parameters, such as biomechanical properties, CCT [31,32,33,34,35], corneal irregularities [36], and corneal refractive surgery [37,38]. Albis-Donado et al. evaluated different external factors that can influence GAT reliability: CCT is crucial, but the authors also emphasized the role of altitude [32]. In fact, they found a higher risk of underestimating IOP not only with thinner corneas, but also with a higher altitude. These outcomes are valid for GAT; on the other hand, dynamic contour tonometry (DCT) seems to be less affected by external factors [32]. Chen et al. demonstrated that CCT influences IOP measured not only with GAT, but also with rebound tonometry and with non-contact tonometry (NCT) [34]; they observed that NCT was mostly influenced by CCT, while GAT was less dependent on CCT.

Regarding corneal abnormalities, Knauf et al. reported that IOP measurement is challenging at various stages of keratoconus (KC), depending on the corneal thickness and biomechanical properties [35]. These features are altered in KC eyes. For similar reasons, IOP is difficult after corneal refractive surgery: changes in corneal thickness and corneal curvature affect IOP measurement reliability with different instruments, and topical steroids used postoperatively may mask the incidence and severity of steroid-induced ocular hypertension in the short term [38].

Since the purpose of this study was to compare IOP measurements in different positions, and GAT cannot be used when a patient is unable to sit at a slit lamp (small children, bed-ridden persons) or in situations such as taking measurements in a standing or supine position, TPA was chosen, among the hand-held IOP measuring devices, because it is one of the most widely used, and so far has not been tested for such a purpose.

## 5. Conclusions

In conclusion, the results of this study confirm the importance of measuring IOP in a supine position, but more importantly suggest that such measurements should also be performed after five minutes in a standing position, as in some jobs (e.g., waiters, policemen, chefs…) people can spend a significant amount of time standing, and this information could be helpful in understanding some cases of optic nerve damage or glaucoma progression, despite an apparently normal IOP.

## Figures and Tables

**Table 1:** Intraocular pressure measurements (in mmHg) in the different positions with a Tono-Pen Avia tonometer.

	Sitting	Supine	ST *	ST-5	Prone **
Mean	14.63	15.93	14.38	15.16	18.15
SD	2.82	3.27	2.96	3.18	3.19
Min	9.50	10.00	9.50	10.00	13.00
Max	22.50	25.50	24.00	24.00	23.00

ST: measurements obtained immediately after assuming a standing position; ST-5: measurements obtained after five minutes in a standing position; SD: standard deviation. * ST data are avilable only for 58 patients; ** prone position data are available only for 23 patients.

**Table 2:** Intraocular pressure difference (in mmHg) measured with a Tono-Pen AVIA tonometer between different positions, with statistical differences.

	Difference ST/Sitting	Difference ST-5/Sitting	Difference Supine/Sitting	Difference ST/Supine	Difference ST-5/Supine
Mean	−0.13	0.53	1.30	−1.43	−0.77
SD	1.63	1.24	1.48	1.74	1.59
95%CI	−0.56/0.30	0.22/0.85	−0.19/1.68	−1.89/−0.97	−1.16/−0.36
*p*	0.548	0.001	<0.001	<0.001	<0.001
	**Difference Prone/Supine**	**Difference** **ST/ST-5**	**Difference** **Prone/ST**	**Difference** **ST-5/Prone**	**Difference Sitting/Prone**
Mean	2.24	−0.67	3.46	−2.46	−3.22
SD	1.92	1.84	2.01	1.67	1.56
95%CI	1.41/3.06	−1.16/−0.19	2.59/4.33	−3.18/−1.73	−3.89/−2.54
*p*	<0.001	0.007	<0.001	<0.001	<0.001

ST: measurements obtained immediately after assuming a standing position; ST-5 min: measurements obtained after five minutes in a standing position; SD: standard deviation; 95%CI: 95% confidence interval around the mean; *p*: level of significance of paired *t*-test.

**Table 3:** Intraocular pressure difference (in mmHg) measured with a Tono-Pen AVIA tonometer between different positions divided by patients’ age, with statistical differences.

A	Difference ST-5/Sitting	Difference ST-5/Supine	Difference ST/ST-5	Difference Supine/Sitting	Difference ST/Sitting	Difference ST/Supine
Mean	0.26	−0.93	−0.79	1.19	−0.55	−1.73
SD	1.26	1.61	2.02	1.60	1.65	1.58
95%CI	−0.17/0.69	−1.48/−0.38	−1.53/−0.05	0.63/1.74	−1.15/0.06	−2.31/−1.15
*p*	0.237	0.002	0.037	<0.001	0.074	<0.001
**B**	**Difference** **ST-5/Sitting**	**Difference** **ST-5/Supine**	**Difference** **ST/ST-5**	**Difference** **Supine/Sitting**	**Difference** **ST/Sitting**	**Difference ST/Supine**
Mean	0.89	−0.56	−0.54	1.44	0.35	−1.09
SD	1.13	1.56	1.63	1.32	1.49	1.87
95%CI	0.44/134	−1.17/0.06	−1.18/0.11	0.92/1.97	−0.23/0.94	−1.83/−0.35
*p*	<0.001	0.075	0.100	<0.001	0.232	0.005

A: patients’ age ≤ 25 years; B: patients’ age > 25 years; ST: measurements obtained immediately after assuming a standing position; ST-5 min: measurements obtained after five minutes in a standing position; SD: standard deviation; 95%CI: 95% confidence interval around the mean; *p*: level of significance of paired *t*-test; in bold: similar level of significance compared to the general population (Table 2).

## Data Availability

The datasets generated and analyzed during the current study are available from the corresponding author upon reasonable request.
